# Detecting causality from short time-series data based on prediction of topologically equivalent attractors

**DOI:** 10.1186/s12918-017-0512-3

**Published:** 2017-12-21

**Authors:** Ben-gong Zhang, Weibo Li, Yazhou Shi, Xiaoping Liu, Luonan Chen

**Affiliations:** 1 School of Mathematics & Computer Science, Wuhan Textile University, Wuhan, 430200 China; 2 Research Center of Nonlinear Science, Wuhan Textile University, Wuhan, 430200 China; 3 Key Laboratory of Systems Biology, Shanghai Institutes for Biological Sciences, Chinese Academy of Sciences, Shanghai, 20031 China

**Keywords:** Causality, Topologically equivalent position, Gene regulations, Short time-series

## Abstract

**Background:**

Detecting causality for short time-series data such as gene regulation data is quite important but it is usually very difficult. This can be used in many fields especially in biological systems. Recently, several powerful methods have been set up to solve this problem. However, it usually needs very long time-series data or much more samples for the existing methods to detect causality among the given or observed data. In our real applications, such as for biological systems, the obtained data or samples are short or small. Since the data or samples are highly depended on experiment or limited resource.

**Results:**

In order to overcome these limitations, here we propose a new method called topologically equivalent position method which can detect causality for very short time-series data or small samples. This method is mainly based on attractor embedding theory in nonlinear dynamical systems. By comparing with inner composition alignment, we use theoretical models and real gene expression data to show the effectiveness of our method.

**Conclusions:**

As a result, it shows our method can be effectively used in biological systems. We hope our method can be useful in many other fields in near future such as complex networks, ecological systems and so on.

## Background

How to correctly detect the causality from the observed time-series is quite important but it is usually very difficult, and has attracted much attention in complex systems in recent years. There are many effective method to infer the causal relation between the variables, such as mutual prediction method [1], state space method [2], phase mode ling method [3], quantifying information method [4], recurrence plots method [5], convergent cross mapping method [6] and so on. As the primary framework, Granger causality (GC) is recognized as one of the most popular measures to reveal causality influence of time-series on the causation problem.

GC can be roughly stated as follows [7]: the variable *X* was said to “Granger cause” *Y* if the predictability of *Y* declines when *X* was removed from the universe of all possible causative variables *u*. The key characteristic of GC is separability, which means that the information for a causative factor only depends on one variable. In other words, if the variable *X* is removed, its information will be eliminated at the same time. However, the assumption of separability is mainly appropriate to the stochastic systems or linear systems because the separability implies that the system is just considered as a part not a whole at one time. Generally, for a linear system with strongly coupled variables, GC is a very powerful tool to detect their interactions. However, it lacks ability to detect the causal relation on weakly coupled variables or nonlinearly coupled variables, in particular for a deterministic system, i.e., for those systems, GC may give ambiguous results or even wrong conclusions.

In order to overcome these drawbacks or shortages, Sugihara et al. [6] proposed a method called convergent cross mapping (CCM) based on the embedded attractor reconstruction. To identify the causal relations between nonlinearly coupled variables or weakly coupled variables, CCM has been shown to have significant advantages over GC, vector autoregression (VAR) [8], conditional mutual information (CMI) [4, 9, 10] and spectral methods [11, 12]. For the topic of causality detection, Hirata et al. [5] also used the recurrence plots method to identify hidden common causes from bivariate time-series, in which a nonconvergent property of a recurrence plot was exploited to deny the existence of causal relation between the bivariate time-series. On the other hand, Hempel et al. [13] proposed and analyzed the inner composition alignment (IOTA) which was permutation-based asymmetric association measure to infer the direction of couplings and indirect links from short time-series. Ma et al. [14] proposed cross map smoothness (CMS) methd to detecting causality with short time series. Runge et al. [15] used the multivariate transfer entropy (TE) method to detect causalities in multivariate time-series. This method can distinguish direct from indirect causality and also identify common drivers. However, it is mainly designed for low dimensional systems. To overcome this limitation, the decomposed transfer entropy was proposed by the same group. Similarly, Runge et al. [16] developed a time-delayed conditional mutual information approach, which is called as momentary information transfer (MIT) and has a well interpretable notion to measure the coupling strength.

The CCM method and many other methods are effective for identifying the causal associations from the observed data, and in particular, CCM method can be thought as another milestone after the GC method to detect causality. However, in spite of those impressive advances on this area, most existing approaches including CCM and GC methods, generally need a long time-series to detect the causal relation, for example, more than 3000 in CCM study. But in many real-world application data, especially in biological systems, the oberved or obtained time-series data (or samples) is usually very short (e.g., sometimes only a few time points). Since these data or samples are highly depended on the experiment or the limited resource. Thus, one natural question is how to detect the causality of these high dimensional short time-series, including those weakly coupled and nonlinearly coupled relations.

In this work, to answer this question, we aim to find an effective method to detect causal relation for high dimensional short time-series or small samples. In other words, in this paper, we propose a new method called topologically equivalent position method shorting for TEP which can detect causality for very short time-series data or small samples. This method is mainly based on attractor embedding theory in nonlinear dynamical systems. Specifically, we exploit the information from the embedding theorem, i.e., two attractors reconstructed from two different observed variables are topological equivalent. That information is used to predict time-series of one variable from another or detect the causality between them based on the principle of topologically equivalent position, i.e., the positions of two corresponding points in the two attractors are topologically equivalent. This prediction can be achieved even by a small number of samples or short time-series. We use both numerical examples and real gene expression data to show the effectiveness of our method. As a result, it shows our method can be effectively used in biological systems. And it can extend GC and CCM methods to general cases. In addition, the comparison studies for different approaches are also provided to show the superiority of our method.

## Methods

### The definition of causality

In dynamical systems theory, the necessary condition that two variables (time-series) are causally linked each other, is that these two variables are from the same dynamical system or they must share the same attractor. This also means that time-series data of one variable contains the information of other variables in the same system or attractor, and thus can be used to predict the dynamics of other variables. Here the attractor means a set of numerical values of the state invariant under the dynamics or the numerical values toward to a system in the course dynamic evolution. Furthermore, according to the Takens’ delay embedding theorem [17], one can use the observed time-series of one variable to reconstruct the original high-dimensional dynamical behavior by lagged-coordinate [18]. In other words, Takens’ delay embedding theorem grantees that data of each variable can reconstruct the attractor of the original (high dimensional) system. Takens’ embedding theorem provides the theoretical foundation for autonomous dynamical systems with noise-free. However, this is not the case in many real systems. Therefore, Stark et al. [19, 20] extend Takens’ embedding theorem to deterministically forced systems (i.e., non-autonomous system) and further they gave the delay embedding theorems for arbitrarily and stochastically forced systems.

**Fig. 1:**
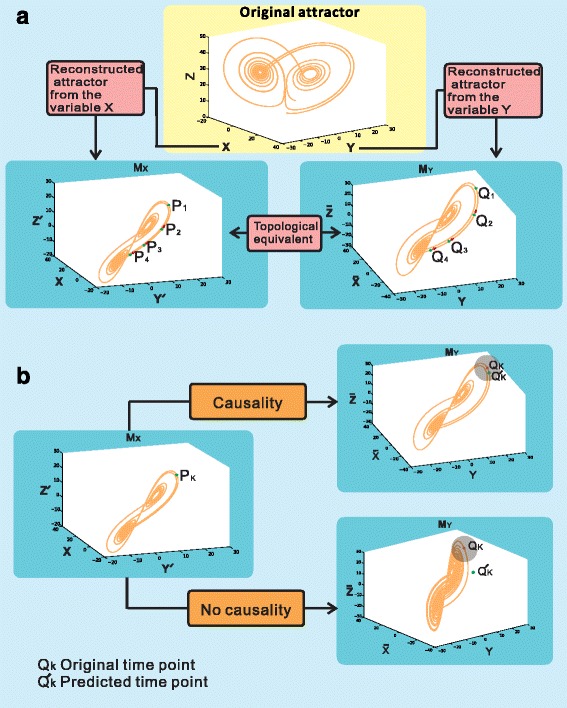
Definition of causality. **a**. the attractors *M*_*X*_ and *M*_*Y*_ are reconstructed from variables *X* and *Y* by lagged-coordinates and they are topological equivalent. **b**. The predicted time-series (points) ${Q}_k^{\prime }\ \left(k=1,2,\cdots \right)$ are located in the nearest neighborhood of *Q*_*k*_ (*k* = 1, 2, ⋯) which implies causality (see the above part). The predicted time-series (points) ${Q}_k^{\prime }\ \left(k=1,2,\cdots \right)$ are outside the nearest neighborhood of *Q*_*k*_ (*k* = 1, 2, ⋯) which implies no causality (see the below part). Here, the nearest neighborhood is measured by a ball with a small radius *r* (see the gray district)

In this paper, based on the lagged-coordinate delay embedding theorem, we develop an effective method to detect the causal relation between a pair of variables (i.e.,genes) for short gene expression data. Specifically, first we define the causality, which is actually a prediction-based concept in this work. We denote the shared common attractor (original attractor in Fig. 1a) as *M* and the reconstructed attractors by lagged-coordinates from their components, for example, *X, Y* as *M*_*X*_, *M*_*Y*_, respectively (Fig. 1a). Based on the embedding theorem, the reconstructed attractors *M*_*X*_ and *M*_*Y*_ are topologically equivalent.

The variable *Y* causes the variable *X* if and only if one can use the information of *X* to predict the variable of *Y.* Taking Fig. 1b for example, two time-series variables {*P*_*i*_}, (*i* = 1, 2, ⋯) and {*Q*_*i*_}, (*i* = 1, 2, ⋯) are located on their corresponding reconstructed attractors *M*_*X*_ and *M*_*Y*_, i.e. *P*_*i*_ ∈ *M*_*X*_, *Q*_*i*_ ∈ *M*_*Y*_, (*i* = 1, 2, ⋯). Now we use the information of *P*_*i*_ (*i* = 1, 2, ⋯) to predict *Q*_*i*_ (*i* = 1, 2, ⋯), if the predicted time-series (samples) ${\overline{Q}}_i\ \left(i=1,2,\cdots \right)$ are in the near neighborhood of *Q*_*i*_ (*i* = 1, 2, ⋯) on attractor *M*_*Y*_, we call that *Y* causes *X* (see Fig. 1b). Otherwise, if the predicted time-series (points) ${Q}_i^{\prime}\left(i=1,2,\cdots \right)$ are outside the near neighborhood of *Q*_*i*_ (*i* = 1, 2, ⋯),we say that *Y* does not cause *X* (see Fig. 1b). Clearly, such causality is based on the prediction of one variable from data of another variable, and thereby it is the prediction-based causality.

#### Topologically equivalent position method

To detect the causality between time-series variables, we propose a new method which we call it topologically equivalent position method shorting for TEP. We will use this method to identify the causal relation for oberved short time-series data or small samples, for which most of existing methods may fail due to insufficient information. This method is based on barycentric coordinates obtained by tessellation [21] which was extended to high-dimensional phase space that can model a high-dimensional time series [22]. We first make basic assumption for our method, i.e., the observed data of variables are from the same system or share a common attractor. Thus, according to the delay embedding theorem, each component of the observed time-series (samples) can reconstruct the topologically equivalent attractor of the original system.

We know that the reconstructed attractors *M*_*X*_ *,M*_*Y*_ are topological equivalent (see Fig. 1a). Here topological equivalent means that the dynamical behavior of the original system is preserved. Next, we first describe TEP as follows.

**Definition 1 (TEP)** For any two points *P*_*i*_ ∈ *M*_*X*_, *Q*_*i*_ ∈ *M*_*Y*_(*i* = 1, 2, ⋯) are called topologically equivalent positions (TEP), if and only if the relative distances from *P*_*i*_, *Q*_*i*_ to any other points on respective attractors *M*_*X*_, *M*_*Y*_ are invariant.

**Fig. 2:**
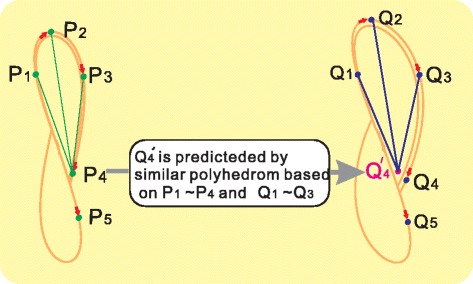
Illustration of topologically equivalent attractors and topologically equivalent position. The two attractors *M*_*X*_ and *M*_*Y*_ are reconstructed from the original system by lagged-coordinates of its components *X* and *Y*. These two reconstructed attractors are topological equivalent. There are two short time-series {*P*_*i*_} and {*Q*_*i*_}(*i* = 1, 2, ⋯) on these two topologically equivalent attractors, respectively. And we call two points as topologically equivalent position, taking *P*_4_ and *Q*_4_ for example, if and only if the relative distance from *P*_4_ and *Q*_4_ to any other points on their corresponding attractors are invariant

To understand this definition, we give the illustration in Fig. 2. In this figure, two points (vectors) on two topologically equivalent attractors *M*_*X*_ and *M*_*Y*_ (taking *P*_4_ and *Q*_4_ for example) are called topologically equivalent position if the following quantities are satisfied:

1 $$ {d}_{4i}=\gamma {D}_{4i},i\le 3, $$

where $\gamma =\frac{d_{12}}{D_{12}},{d}_{12}$ and *D*_12_ are the Euclidean distances of the first two points on their attractors *M*_*X*_ and *M*_*Y*_. *d*_4*i*_ and *D*_4*i*_ are Euclidean distances from points *P*_4_ and *Q*_4_ to other points on their respective attractors *M*_*X*_ and *M*_*Y*_. For a general case, any two points on two topologically equivalent attractors are called topologically equivalent position, if they satisfy

2 $$ {d}_{ij}=\gamma {D}_{ij},i\ge 3,j=1,2,\cdots, i>j, $$

where *γ* is a constant.

We also assume that the relative position of points *P*_*i*_, *Q*_*i*_(*i* = 1, 2, ⋯) on the reconstructed attractors *M*_*X*_, *M*_*Y*_ are known. Next, we use the information defined in (2) to detect causal relation between these two time-series from the topologically equivalent attractors.

In order to identify the causality between two time-series from their topologically equivalent attractors, first we use the information of *P*_1_ ∼ *P*_*i*_ and *Q*_1_ ∼ *Q*_*i* − 1_ to predict ${Q}_i^{\prime }$. For example, we use *P*_1_ ∼ *P*_4_ and *Q*_1_ ∼ *Q*_3_, to predict ${Q}_4^{\prime }$,where ${Q}_4^{\prime }$ is decided by similar polyhedron based on *P*_1_ ∼ *P*_4_ and *Q*_1_ ∼ *Q*_3_ by using (2) . The next important step is to evaluate this prediction. Our criterion is to check the error between the predicted point ${Q}_4^{\prime }$ and the real point *Q*_4_. We denote the error as

3 $$ \epsilon =\left|{Q}_4^{\prime }-{Q}_4\right|. $$

If the error *ϵ* is sufficiently small (less than 10^−3^), it implies an accurate prediction from *P*_4_ to *Q*_4_. In the same way, we can check other points until all the points are evaluated. Finally, we obtain the mean error or the total error. If they are sufficiently small, it means that the information of {*P*_*i*_}, (*i* = 1, 2, ⋯) can predict {*Q*_*i*_}, (*i* = 1, 2, ⋯). This also implies that {*P*_*i*_}, (*i* = 1, 2, ⋯) has strong relationship with {*Q*_*i*_}, (*i* = 1, 2, ⋯). In other words, the error can reflect the causal relation between these two variables *X* and *Y*. Clearly, even three points are sufficient to estimate the TEP between two time-series in theoretically (to produce ‘two distances’ needs three points at least), which is a major advantages of this method.

However, to directly evaluate the error *ϵ* of Eq. (3) is not a trivial problem. In particular, for a high dimensional system, it is very difficult to calculate the predicted point *Q*_4*i*_ because we need to solve a large number of nonlinear equations. Here, instead of the error *ϵ* of Eq. (3), we evaluate the following relative error.

4 $$ {\varepsilon}_{ij}=\frac{\left|{r}_{ij}/{r}_{12}-{D}_{ij}/{D}_{12}\right|}{D_{ij}/{D}_{12}},i\ge 3,i>j,j=1,2,\cdots, $$

where *r*_*ij*_ is the distance from the predicted points to the real data points. Next, we show that it is not necessary to calculate the predicted points for the error evaluation. From Fig. 2, we know *r*_12_ = *d*_12_ *.* Therefore, *ε*_*ij*_ can rewritten as

5 $$ {\varepsilon}_{ij}=\frac{\left|{r}_{ij}-\gamma {D}_{ij}\right|}{\gamma {D}_{ij}}, $$

Clearly, we substitute *Q*_*i*_ into (5), i.e., substitute *d*_*ij*_ into (5), then the error *ε*_*ij*_ can be obtained without solving ${Q}_i^{\prime }$. Since *d*_*ij*_ and *D*_*ij*_ is known, it is easy and straightforward to calculate the relative error *ε*_*ij*_ *.* Therefore, small error *ε*_*ij*_ implies that the predicted ${Q}_i^{\prime }$ is in the near neighbor of *Q*_*i*_ or is accurate.

We further scale the error *ε*_*ij*_ by the exponential function so that the error is normalized between 0 and 1. Therefore, the final score of a pair of observed time-series is defined as:

6 $$ \varepsilon =\frac{1}{n-1}{\sum}_{i=3}^n\left(\frac{1}{i-1}{\sum}_{j=1}^{i-1}\frac{1}{\mathit{\exp}\left({\varepsilon}_{ij}\right)},i\ge 3,i>j,j=1,2,\dots, \right) $$

where *n* is the number of the time points (samples) for error estimation. Generally, we use leave-one-out scheme to evaluate all the observed time points (samples).

By using this score function, we identify the causal relation both for numerical examples and real gene expression data in next two sections.

**Remark:** Both the CMS method used in [14] and TEP method proposed here used the delay embedding theory for a nonlinear dynamical system but their idea is different. On one hand, the key point of CMS method is to construct ‘Smoothness Map’. The key idea of our TEP method is to obtain barycentric coordinates by tessellation. On the other hand, the CMS method used nerual network to train the data to show whether the cross map can map the nearest neighbors to mutual neighbors. So that it can be used to detect causality (see Fig. 1a-b in [14]). Our TEP method use the relative distances to predict the next time point and then calculate the error between the predicted point and real point (see Fig. 2). By using this to detect the causal relation between two time series or small samples.

## Results

To validate the effectiveness of our TEP method, we first apply our TEP method to both several benchmark examples and gene expression data. The theoretical models used here were the same ones used in [6].

### Causal relation of logistic difference equations

The first example is logistic difference equations. Since we know the underlying relations between the variables in advance, we just use these mathematical models to identify the validity of our proposed method. Considering the following two coupled Logistic difference equations which exhibit chaotic behavior [23]

7 $$ \left\{\begin{array}{c}X\left(t+1\right)=X(t)\left[{r}_x-{r}_xX(t)-{\beta}_{x,y}Y(t)\right],\\ {}Y\left(t+1\right)=Y(t)\left[{r}_y-{r}_yY(t)-{\beta}_{y,x}X(t)\right].\end{array}\right. $$

with *r*_*x*_ = 3.8*, r*_*y*_ = 3.5*, β*_*x*, *y*_ = 0.02*, β*_*y*, *x*_ = 0.1*,* and the initial conditions *X*(1) = 0.4*, Y* (1) = 0.2*.*

**Fig. 3:**
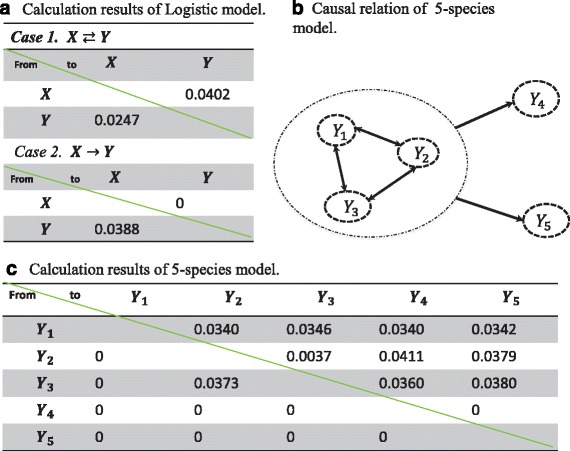
The results of the numerical examples by our method. **a**. The results of Logistic model by our method (rows → columns). **b**. The real interaction of a 5-species model. In this model, *Y*_1_, *Y*_2_ and *Y*_3_ are coupled each other, and also *Y*_1_, *Y*_2_ *, Y*_3_ drive *Y*_4_ and *Y*_5_ *.* However, *Y*_4_ and *Y*_5_ do not have any effect on *Y*_1_, *Y*_2_ and *Y*_3_ *.* **c**. The results of the five species model by our method (rows → columns). Here 0 means that there is no causal relation

By using the TEP method, we first check the bidirectional causal relation and then the unidirectional causal relation of the above system (7) between the variables *X* and *Y*. Since the two cases *β*_*y*, *x*_ = 0 or *β*_*x*, *y*_ = 0 are equivalent, without loss of generality, here we consider the case *β*_*y*, *x*_ = 0. The system (7) becomes

8 $$ \left\{\begin{array}{c}X\left(t+1\right)=X(t)\left[{r}_x-{r}_xX(t)-{\beta}_{x,y}Y(t)\right],\\ {}Y\left(t+1\right)=Y(t)\left[{r}_y-{r}_yY(t)\right].\end{array}\right. $$

with the same parameters *r*_*x*_ = 3.8*, r*_*y*_ = 3.5*, β*_*x*, *y*_ = 0.02*,* and the initial conditions *X*(1) = 0.4*, Y* (1) = 0.2*.* We give the calculation results by using our method shown in Fig. 3a.

Comparing the results in Fig. 3a with those in (7) and (8), clearly our method can identify the causal relation of the two dimensional difference Logistic model correctly. And also comparing with the results in [6], we use much less time points (actually only 10 time points) to identify the causal relation of the logistic model.

### Causal relation of 5-species mathematical model

To further verify the effectiveness of our TEP method, we detect the causal relation between the variables of a 5-species model. The model can be described by the following system shown in Fig. 3b.

9 $$ \left\{\begin{array}{c}{Y}_1\left(t+1\right)={Y}_1(t)\left[4-4{Y}_1(t)-2{Y}_2(t)-0.4{Y}_3(t)\right],\\ {}{Y}_2\left(t+1\right)={Y}_2(t)\left[3.1-0.3{Y}_1(t)-3.1{Y}_2(t)-0.93{Y}_3(t)\right],\\ {}{Y}_3\left(t+1\right)={Y}_3(t)\left[2.12+0.636{Y}_1(t)+0.636{Y}_2(t)-2.12{Y}_3(t)\right],\\ {}{Y}_4\left(t+1\right)={Y}_4(t)\left[3.8-0.111{Y}_1(t)-0.011{Y}_2(t)+0.131{Y}_3(t)-3.8{Y}_4(t)\right],\\ {}{Y}_5\left(t+1\right)={Y}_5(t)\left[4.1-0.082{Y}_1(t)-0.111{Y}_2(t)-0.125{Y}_3(t)-4.1{Y}_5(t)\right].\end{array}\right. $$

From Fig. 3b, it is clear that *Y*_1_, *Y*_2_ and *Y*_3_ are coupled each other, and also *Y*_1_, *Y*_2_, *Y*_3_ drive *Y*_4_ and *Y*_5_. However, *Y*_4_ and *Y*_5_ do not have any effect on *Y*_1_, *Y*_2_ and *Y*_3_. It agrees with our calculation results (by using 15 time points) listed in Fig. 3c.

Both these two simple models show that our method works well by using a small number of samples, i.e., it can detect the causality between the variables correctly.

### *E. coli* Gene expression data

In this section, we apply our method to detect the causal gene regulation between a pair of genes. The gene regulatory network used here is the bacterium *E.Coli,* as described in [24]. It has been shown that 100 genes can approximate significantly well the statistical properties of the whole network [13, 25]. In order to make comparison, here we also analyze a subnetwork with 100 genes where every gene represents a node and the dynamics of each node (gene) is described by Michaelis-Menten and Hill kinetics. We should point out that for more genes which means the dimensional is much more higher, it still can be disposed by our method. The only thing is that it needs much more time to calculate the errors. Moreover, gene expression data with 10 time points are measured as the same used in [13, 25].

**Fig. 4:**
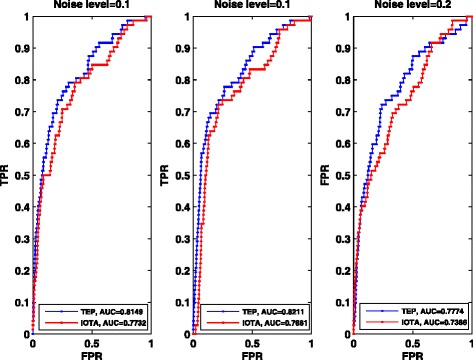
ROC curves of the *E. coli* network with 100 genes with different noise levels. IOTA represents the method used in [13], and TEP is our method used in this paper

By using the algorithm above, we first calculate *ε* for each pair of genes. Therefore, there are totally $$ {P}_{100}^2, $$ *i*. *e*. , 9900 *εs*. We also use the receiver operating characteristics (ROC) curves with different noise level, i.e., noise free, noise level 0.1 and 0.2, respectively. At the same time we also compare our method with the IOTA method used in [13]. The comparing results are shown in Fig. 4. In addition, to evaluate and rank the overall performance, we provide the area under ROC curves (AUC), and the results are shown in Table 1.

**Table 1:** Summary of AUC for *E. coli* networks

Noise level	AUC for TEP	AUC for IOTA
0	0.8149	0.7732
0.1	0.8211	0.7681
0.2	0.7774	0.7386

From the ROC curves above and the statistic analysis of ROC curves, clearly, TEP is effective to detect the causality of gene regulations for the observed or obtained short time-series data. Comparison results between our method and IOTA method (see Fig. 4) also demonstrate the superiority of TEP on the accuracy.

### Yeast gene expression data

**Fig. 5:**
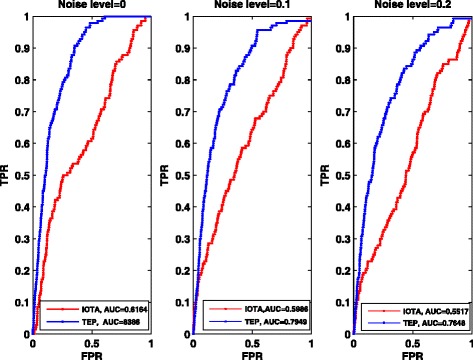
ROC curves of the Yeast network with 100 genes with different noise levels. IOTA represents the method used in [13], and TEP is our method used in this paper

Now we detect the causal gene regulations from yeast gene expression data with 10 time points. The network structures were downloaded from the reference [26, 27]. Just like the *E.coli* gene expression data, here we first conduct the statistics analysis of ROC curves. At the same time, we compare our method with the IOTA method. The results are shown in Fig. 5 and Table 2, which validated the effectiveness of our method.

**Table 2:** Summary of AUC for yeast networks

Noise level	AUC for TEP	AUC for IOTA
0	0.8386	0.6164
0.1	0.7949	0.5986
0.2	0.7648	0.5517

### Rat circadian rhythm gene expression data

Circadian rhythm is fundamentally important for mammals in their physiological processes. To identify the important circadian genes and their roles in their relevant processes is important to elucidate their mechanism of rhythms, in particular, at a network level. In fact, there exists many key circadian genes and functional organization interaction, which generate circadian oscillations. Based on the rat circadian rhythm gene expression data [28], we detect the causal relations among genes by our method.

**Fig. 6:**
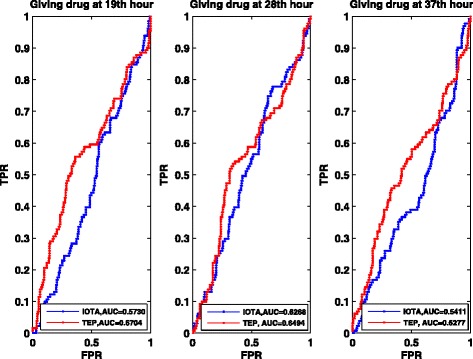
ROC curves of the Rat circadian rhythm with 18 key related genes by applying drug at different time points. IOTA represents the method used in [13], and TEP is our method used in this paper

For circadian rhythm related genes [29, 30], there are 18 key circadian genes identified in mammals and also extensively studied. We further add 22 circadian related genes which all have protein interactions and phosphorylations relationships with the 18 key circadian genes. In other words, we mainly study the causal relations among 40 genes by using the gene expression data. The detail function relationship can be found in [28] (Fig. 2 in [28]). Figure 6 and Table 3 show the ROC curves and the AUC as well as the comparison result, which are obtained based on the gene expression data with 18 time points as the same case in [28].

**Table 3:** Summary of AUC for circadian rhythm networks

Time points of using drug	AUC for TEP	AUC for IOTA
19th hour	0.6704	0.5730
28th hour	0.6494	0.6268
37th hour	0.6277	0.5411

**Fig. 7:**
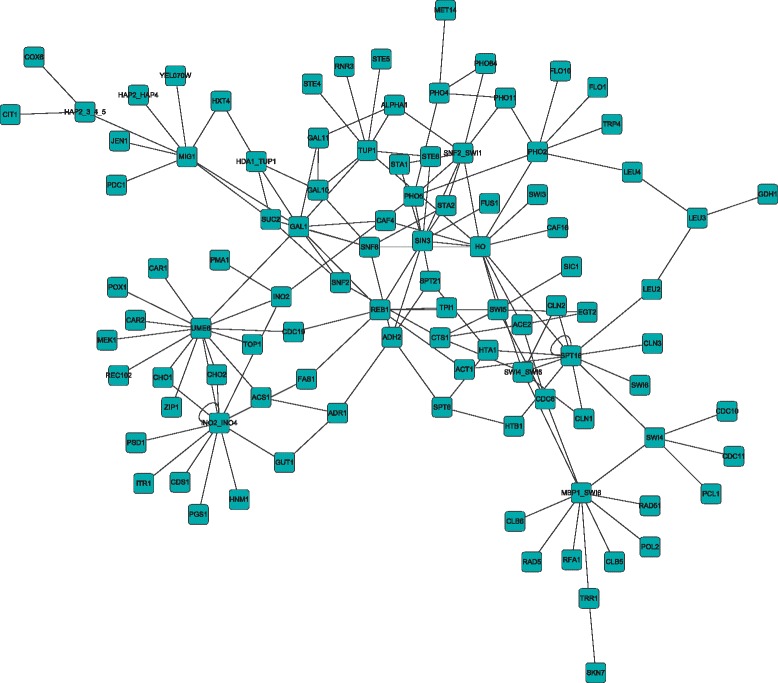
The real gene regulatory network of E.Coli with 100 genes

**Fig. 8:**
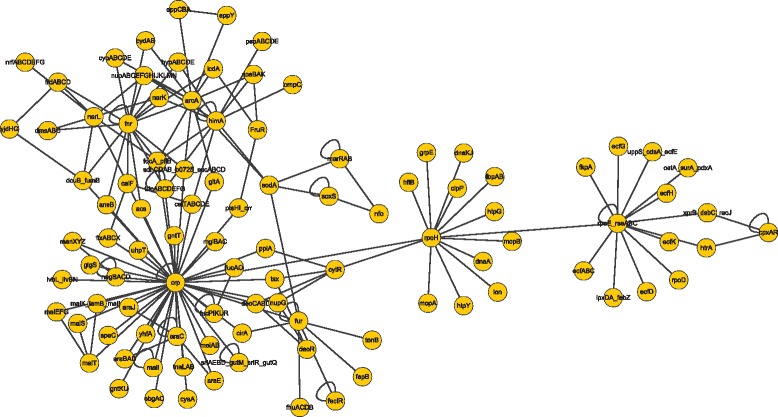
The real gene regulatory network of Yeast with 100 genes

In order to make our results more clearly, we give the real gene regulatory network of 100 genes of *E. coli* and Yeast in Figs. 7 and 8. The results of the above three gene expression data show that TEP method works well even with a small number of samples. Comparing with the IOTA method, the truth positive rate (TPR) are higher with the same false positive rate (FPR), which means that our method is effective to detect the causal relation than IOTA method.

## Discussion

How to detect the causal relations from short time-series data is really very important. Especially for the genes, because the obtained causal relations among the genes can provide valuable information and insights into topological structures of gene regulatory networks. Besides the gene regulatory networks, our method can be used in many other complex networks. However, we must also point out that there still exist false predictions, e.g., many false prediction by the circadian rhythm gene expression data. As a future topic, we will study the dependence of our method on the data and its length.

## Conclusion

In this work, a new method which called topologically equivalent position method is proposed. It is a prediction-based method. It can be effectively used to detect the causality of the observed short time-series data or very small samples. Both the numerical examples and gene expression data have been used to validate the proposed method. Different from the existed method, such as Granger causality and CCM, our method not only is simple in terms of computational procedure, but also can be applied to nonlinear systems. The most important is that it can identify the causality for the observed observed time-series just from short time points. This is very useful for real-world data, in particular, the gene expression data, which are typically very short (≈10 points).

## References

[1] Schiff SJ, So P, Chang T, Burke RE, Sauer T (1996). Detecting dynamical interdependence and generalized synchrony through mutual prediction in a neural ensemble. Phys Rev E.

[2] Arnhold J, Grassberger P, Lehnertz K, Elger CEA (1999). Robust method for detecting interdependences: application to intracranially recorded EEG. Physica D.

[3] Rosenblum M, Pikovsky A (2001). Detecting direction of coupling in interacting oscillators. Phys. Rev. E.

[4] Schreiber T (2000). Measuring information transfer. Phys Rev Lett.

[5] Hirata Y, Aihara K (2010). Identifying hidden common causes from bivariate time-series: a method using recurrence plots. Phys Rev E.

[6] Sugihara G, May R, Ye H, Hsieh C, Deyle E, Fogarty M, Munch S (2012). Detecting causality in complex ecosystems. Science.

[7] Granger CWJ (1969). Investigating causal relations by econometric models and cross-spectral methods. Econometrica.

[8] Engle RF, Granger CWJ (1987). Co-integration and error correction: representation, estimation, and testing. Econometrica.

[9] Hiemstra C, Jones JD (1994). Testing for linear and nonlinear granger causality in the stock price-volume relation. J. Finance.

[10] Faes L, Nollo G, Porta A (2011). Information-based detection of nonlinear granger causality in multivariate processes via a nonuniform embedding technique. Phys Rev E.

[11] Ding M, Chen Y, Bressler SL (2006). Handbook of time-series analysis.

[12] Dhamala M, Rangarajan G, Ding M (2008). Estimating granger causality from Fourier and wavelet transforms of time-series data. Phys Rev Lett.

[13] Hempel S, Koseska A, Kurths J, Nikoloski Z (2011). Inner composition alignment for inferring directed networks from short time-series. Phys Rev Lett.

[14] Ma H, Aihara K, Chen L (2014). Detecting causality from nonlinear dynamics with short-term time series. Sci Rep.

[15] Runge J, Heitzig J, Petoukhov V, Kurths J (2012). Escaping the curse of dimensionality in estimating multivariate transfer entropy. Phys Rev Lett.

[16] Runge J, Heitzig J, Marwan N, Kurths J (2012). Quantifying causal coupling strength: a lag-specific measure for multivariate time-series related to transfer entropy. Phys Rev E.

[17] Takens F (1981). Detecing strange attractors in turbulence, lecture notes in mathematics Vol. 898, edited by D. A. Rand and L. S. Young.

[18] Sauer T, Yorke JA, Casdagli M (1991). Embedology. J Stat Phys.

[19] Stark J (1999). Delay embeddings for forced systems I. Deterministic forcing. J. Nonlinear Sci..

[20] Stark J, Broomhead DS, Davies ME, Huke J (2003). Delay Embeddings for forced systems. II. Stochastic forcing. J Nonlinear Sci.

[21] Mees A (1991). Int. dynamics systems and tesselations: detecting determinism in data. J. Bifurcation. Chaos.

[22] Hirata Y, Shiro M, Takahashi N, Aihara K, Suzuki H, Mas P (2015). Approximating high-dimensional dynamics by barycentric coordinates with linear programming. Chaos.

[23] Lloyd AL (1995). The coupled logistic map: a simple model for the effects of spatial heterogeneity on population dynamics. J Theor Biol.

[24] Shen-Orr SS, Milo R, Mangan S, Alon U (2002). Network motifs in the transcriptional regulation network of Escherichia Coli. Nat Genet.

[25] Bulcke TV, Leemput KV, Naudts B (2006). SynTReN: a generator of synthetic gene expression data for design and analysis of structure learning algorithms. BMC Bioinformatics.

[26] Van den Bulcke T, Van Leemput K, Naudts B, van Remortel P, Ma H, Verschoren A, De Moor B, Marchal K. SynTRen generator, version 1.1.3. 2006. http://homes.esat.kuleuven.be. 10.1186/1471-2105-7-43 PMC1373604 16438721

[27] Guelzim N, Bottani S, Bourgine P, Kepes F (2002). Topological and causal structure of yeast transcriptional regulatory network. Nat Genet.

[28] Wang Y, Zhang XS, Chen LA (2009). Network biology study on circadian rhythm by integrating various OMICS data. OMICS: a journal of. Integr Biol.

[29] Ueda HR, Hayashi S, Chen W, Sano M, Machida M, Shigeyoshi Y (2005). System-level identification of transcriptional circuits underlying mammalian circadian clocks. Nat Genet.

[30] Ko CH, Takahashi JS (2006). Molecular components of the mammalian circadian clock. Hum Mol Genet.

